# Clinician’s Experience of Working with an Intensive Outpatient Programme for Child and Adolescent Eating Disorders—A Reflexive Thematic Analysis

**DOI:** 10.3390/bs16020276

**Published:** 2026-02-14

**Authors:** Cliona Rae Brennan, Ellen McAdams, Elena Pears, Amy Chimes, Anna Konstantellou, Mima Simic, Julian Baudinet

**Affiliations:** 1 Maudsley Centre for Child and Adolescent Eating Disorders, South London and Maudsley NHS Trust, Denmark Hill, London SE5 8AZ, UK; 2 Department of Nutritional Sciences, Franklin-Wilkins Building, Kings College London, London SE1 9NQ, UK; 3 Department of Nutrition and Dietetics, London Metropolitan University, Holloway Road, London N7 8DB, UK; 4 Centre for Research in Eating and Weight Disorders (CREW), Institute of Psychiatry, Psychology and Neuroscience, King’s College London, De Crespigny Park, London SE5 8AZ, UK

**Keywords:** eating disorders, intensive outpatient programme, adolescence, family-focused treatment

## Abstract

Although intensive outpatient programmes (IOPs) are becoming more prevalent, the evidence base, particularly within the UK, remains limited. Given clinicians’ central role in developing, delivering, and adapting these emerging models of care, their perspectives are essential to understanding how IOPs function in practice. This study therefore aims to address a significant gap in the literature by exploring clinicians’ experiences of working with an IOP and the strengths and opportunities arising from this. Fifteen experienced clinicians participated in individual semi-structured interviews after working with the IOP. Open-ended questions guided the discussions, which were recorded and transcribed verbatim. Data were analysed using the six stages of reflexive thematic analysis. The analysis generated three key themes: (1) Tri-directional Collaboration, (2) Creating Space for Change, and (3) Transitions as Turning Points. Clinicians felt that the IOP provided a structure that strengthened and reinforced the therapeutic alliance between parents and clinicians, helped arrest rapid deterioration, and created space for thoughtful planning. Embedding IOPs within stepped-care frameworks may offer an effective and scalable means of expanding system capacity while delivering enhanced, flexible support during periods of heightened risk. However, longitudinal, mixed-methods evaluations are needed to clarify the sustainability of progress post-IOP and to identify predictors of positive transitions.

## 1. Introduction

Eating disorders (EDs) are diverse and complex psychiatric conditions (Bryant-Waugh & Baudinet, 2025; Withnell et al., 2022). They carry some of the highest rates of morbidity and mortality among psychiatric illnesses (Keshaviah et al., 2014; Matthews et al., 2022). The prevalence and burden of EDs has risen over recent decades (Wu et al., 2020), with a significant surge identified during the COVID-19 pandemic (Taquet et al., 2022), leading to ongoing discussions around care pathways and treatment settings (Ali et al., 2021; Cini & Salucci, 2024).

Evidence highlights the importance of early identification, rapid assessment, and timely initiation of treatment, with shorter duration of untreated illness associated with improved outcomes and a greater chance of full recovery (Eisler et al., 2016; Madden, 2015; Stewart et al., 2022). Longer duration of untreated illness can result in increased maladaptive behaviours accompanied by entrenched and habitual disordered thought patterns (Mills et al., 2023). Across treatment settings, early nutritional rehabilitation, particularly in restrictive EDs, remains a central prognostic indicator, supported by structured eating routines and the active involvement of parents and carers (Mairs & Nicholls, 2016; National Institute of Health and Care Excellence, 2017).

Treatment for EDs takes place across a wide continuum of care including outpatient care, intensive outpatient programmes (IOP) (Ali et al., 2021), day programmes (Baudinet & Simic, 2021) or partial hospital programmes (Van Huysse et al., 2022), residential programmes, and inpatient hospitalisation (Isserlin et al., 2020) (see Table 1 for details). Factors such as illness severity, medical and psychiatric risk, motivation, and engagement in treatment plans impact decisions on location of care. Movement across levels of care can be bidirectional, with graded intensity offering step up or step down, dependant on clinical need. Although guidance recommends that treatment take place in the community, inpatient settings may offer benefits for young people when community options have not achieved symptom improvement (Anderson et al., 2017; Herpertz-Dahlmann et al., 2021).

Outpatient community treatment is the preferred approach, enabling young people to remain within their home environment and support networks (National Institute of Health and Care Excellence, 2017). Community intensive treatment has been shown to support good clinical outcomes, as well as reduced financial burden across research studies (Ali et al., 2021; Buchman et al., 2019; Simic et al., 2018). Although standard outpatient treatment for ED in young people often yields favourable outcomes in ED symptomology, additional input can be required to prevent inpatient admission. Intensified community options provide increased support within a community context (BEAT, 2015; Datta et al., 2020; Madden, 2015), delivering multi-disciplinary medical, psychological, and nutritional interventions and enabling young people to remain at home—reducing disruption to education, peer relationships, and other developmental domains (Colla et al., 2023; Gledhill et al., 2023, 2025; Herpertz-Dahlmann et al., 2021). IOPs have emerged as a flexible and responsive addition to the continuum of care. In the UK, IOPs represent a distinct and increasingly important model of ED care that differs conceptually and structurally from traditional day programmes. Unlike day programmes, which typically involve months of group-based, full-day treatment, UK IOPs are brief (usually weeks rather than months), highly individualised interventions, with care delivered primarily on a one-to-one basis to the young person and family and minimal or no reliance on group therapy. Their core function is to provide rapid, flexible, and responsive outpatient support, often with little or no waiting time, for individuals at imminent risk of hospital admission, while enabling them to remain embedded in their everyday environments (Ali et al., 2021; Baudinet & Simic, 2021; Isserlin et al., 2020). IOPs also frequently function as step-down care, supporting earlier discharge from inpatient or day programmes and facilitating smoother transitions back to routine outpatient care.

This UK model differs from many international, and particularly US IOPs which are often less intensive and structured as group-based programmes delivered over several half-days per week, conceptually closer to modified day programmes (Herpertz-Dahlmann, 2021). IOPs typically adopt a formulation-driven, family-based approach, drawing on principles used across outpatient and day programme-treatment modalities (Baudinet et al., 2021a, 2021b; Fisher et al., 2023).

There are many factors that can improve the outcomes of ED treatment, with the therapeutic alliance between the health professional and the individual being consistently associated with favourable outcomes (Zaitsoff et al., 2015). Therapeutic alliance is closely linked to engagement, early weight gain, and broader symptom change, with particularly complex and bidirectional processes observed in anorexia nervosa (Pereira et al., 2006; Werz et al., 2025). The development of a strong therapeutic alliance is not solely a function of treatment structure but is actively shaped by clinicians’ relational skills, clinical judgement, and capacity to work collaboratively with families and multi-disciplinary teams.

Positioned between outpatient and day programme care, IOP may offer distinct relational conditions that influence how therapeutic alliance and engagement are established and maintained. Clinicians working alongside IOPs are uniquely placed to observe how factors such as treatment intensity, continuity of contact, and opportunities for family and team collaboration affect alliance development and early engagement. As such, clinicians’ experiential insights are essential for understanding the specific contributions of IOPs within the continuum of ED care, including whether and how this treatment modality enhances, alters, or challenges the formation of therapeutic alliance. Despite the increasing prevalence of IOPs (Cini & Salucci, 2024), particularly in the UK, these clinician perspectives remain underrepresented in the literature. This study therefore seeks to address this gap by exploring clinicians’ experiences of working within an IOP, with a focus on how this setting shapes therapeutic relationships, engagement, and perceived treatment impact.

## 2. Methods

### 2.1. Ethical Considerations

Approval for this project was granted by South London and Maudsley Child and Adolescent Mental Health Services (CAMHS) service evaluation and audit committee on 4 March 2025 (Reference: #708). Informed consent was gained from all participants.

### 2.2. Sample

Various methods for sample size calculation in qualitative research exist (Fugard & Potts, 2015). Data saturation is one method that has been proposed to calculate sample size (Guest et al., 2020); however, this method has been disputed by some authors (Braun & Clarke, 2021). Alternative proposed guidelines for thematic analysis, proposed by Braun and Clarke, categorise sample size suggestions by the type of data collection and the size of the project. For small projects, 6–10 participants are recommended for individual interviews (Braun & Clarke, 2012). Sample size in this study was calculated using the guidelines suggested above by Braun and Clarke (i.e., ideal sample size estimate of at least 10).

All clinicians working in the Maudsley Centre for Child and Adolescent Eating Disorders (MCCAED) service who had experience of working with the IOP were invited to participate.

Clinicians were contacted post working collaboratively with the IOP and invited to take part in an online interview. Participation was fully voluntary, with no incentives provided for taking part. Interviews were facilitated by an independent interviewer, with no prior experience of working in the IOP. All participants were qualified and experienced in varying therapeutic treatments such as Family Therapy for Anorexia Nervosa (FT-AN) and Bulimia Nervosa (FT-BN), holding roles as family therapists, psychiatrists, nurse therapists, dietitians or clinical psychologists. Participants were included if they had worked with the IOP team with at least one shared patient. Exclusion criteria were not being a clinical member of staff and not having experience working with the IOP.

### 2.3. Programme Description

The IOP in this study is for children and adolescents under 18 years of age with an ED. The IOP accepts referrals for all restrictive ED diagnoses, including AN, BN, Avoidant Restrictive Food Intake Disorder (ARFID), and atypical ED, that may result in physical health deterioration. It was developed within the specialist outpatient Maudsley Centre for Child and Adolescent Eating Disorders (MCCAED) at the Maudsley Hospital in 2024. The IOP is not a standalone treatment, but is embedded within a comprehensive outpatient ED service that has expertise in ED-focused family therapy (Eisler et al., 2016), multi-family therapy (Baudinet & Eisler, 2024), and other evidence-based treatments for ED. The IOP intervention is individualised to each family and based off the collaborative formulation developed (Baudinet et al., 2021a). Sessions are provided by a multi-disciplinary team that is equipped to manage severe physical and psychiatric risk. In MCCAED, the IOP is offered to young people who are at imminent risk of becoming medically unstable and being admitted to hospital.

Young people are referred to the IOP if there is a deterioration in physical health parameters set out by UK ED national guidance (Royal College of Psychiatrists, 2022). IOP aims to work alongside the outpatient care coordinator in helping the young person and their family to arrest deterioration in health and support them to remain in outpatient treatment, as opposed to moving onwards to inpatient care. Regular contact between the family, IOP, and referring clinician continues throughout the IOP period. This includes weekly joint reviews with the referring clinician, family, and IOP team to discuss progress with the IOP team, any issues emerging, and review short term goals. Goals typically revolve around stabilisation and hospital avoidance. If the young person is already admitted to hospital for physical stabilisation and refeeding, joint reviews will still occur; however, the reviews focus on supporting safe discharge back into the community. Discharge from IOPs is generally back to outpatient treatment. Young people who continue to deteriorate and are unsafe to continue in outpatient care are referred for an inpatient admission.

Prior to commencing the IOP most young people express low motivation to recover, and struggle to engage with the outpatient treatment offered, have multiple maintaining factors for restrictive ED, and their parents may have been struggling to provide consistent support at home. Dietary restriction leading to ongoing weight loss and physical symptoms of malnutrition are common in most patients.

The IOP team comprises nurses, a psychiatrist, an assistant psychologist, and a dietician. The duration of the programme is four weeks, Monday through Friday, with flexibility to shorten or lengthen treatment where clinically needed. The IOP intensity of attendance is decided on a case-by-case basis according to clinical need. Most young people begin the IOP on a full-time basis with a clear plan of reducing days in the IOP each consecutive week.

IOP treatment begins with daily face-to-face sessions in the first week and gradually transitions to less frequent contact, including virtual or telephone check-ins by the final week. The team adopts a family-based approach to treatment, with a primary focus on physical health restoration (e.g., weight gain). This is tailored to meet the individual needs of each young person, often intensifying the support already being provided by the outpatient eating disorder service.

Common themes for IOP sessions include practical meal support and planning, parent coaching, goal setting, motivational work, distress tolerance skills, and enhanced physical monitoring; all with the overarching aim of preventing or reducing the length of hospital admission. A joint session takes place between the family, IOP clinician, and outpatient therapist at least once each week to establish and review shared goals for IOP, review treatment progress, and collaboratively plan following weeks treatment.

### 2.4. Data Collection

Clinicians experience of IOP treatment was explored using semi-structured interviews conducted remotely via Microsoft Teams. One interviewer and one participant were present for each interview. Interviews were conducted by authors AC and AK and lasted between 30 and 45 min, organised at a time convenient to the participant. Both interviewers worked within the MCCAED team and were trained in conducting semi-structured interviews. All interviews were audio-recorded and transcribed verbatim using Microsoft Teams’ embedded automatic transcription software (version 25306.804). Prior to analysis, all transcripts were anonymised to remove identifiable information. Transcriptions were not provided to any participants for review.

A semi-structured interview schedule was developed collaboratively by CB, EMC, and JB. Clinicians were informed at the beginning of their interview that the aim was to explore their experience of IOP treatment. All clinicians responded to the same set of open-ended questions designed to elicit detailed descriptions of their experiences. Questions covering key areas such as understanding of the IOP purpose; perceptions of intervention intensity; goal setting with the team; the therapeutic alliance; cultural inclusivity; communication and collaboration; the transition out of IOP; and reflections on what aspects of care were helpful or could be improved. Data collection took place between May 2024 and August 2025.

### 2.5. Analysis Plan

The six phases of reflexive thematic analysis as outlined by Braun and Clarke (2012) were used. This process began with an initial period of data immersion. The responses and comments were coded from the open-ended questions to generate preliminary themes. After reviewing and developing these themes, they were defined through reflexive engagement with the data. Three authors (CB, EP, and EMC) were involved in this process. Member checking took place to ensure that themes accurately reflected the team’s views, they were cross-checked with survey respondents and incorporated their feedback to make any necessary adjustments. This aimed to enhance the trustworthiness, credibility, and rigour of the findings.

### 2.6. Reflexivity Statement

CB is a cisgender white female dietitian, EP is a cisgender white female nurse, and EMC is a cisgender white female assistant psychologist—all working within the MCCAED team. They understand the inherent biases that their professional roles bring to the survey questions and the themes drawn from the responses. Their alignment with the therapeutic models used within MCCAED and professional backgrounds may have shaped theme construction. The authors acknowledge that their perspectives influence the results and conclusions, and they have considered these biases in their analysis. Three authors with differing levels of experience and professional backgrounds were involved in the analysis to promote reflection on inherent biases that exist given their own perspectives and experiences.

## 3. Results

### 3.1. Sample

Twenty-one referring clinicians were invited to participate. Fifteen took part, and these were from a range of professional backgrounds, including nurses (*n* = 3), family therapists (*n* = 5), dietitians (*n* = 1), psychologists (*n* = 4), and psychiatrists (*n* = 2) representing the multi-disciplinary team in MCCAED.

### 3.2. Qualitative Data

Three themes were generated and understood in the context of the referring clinicians’ experience of working with the IOP (Please see Table 2).

#### 3.2.1. Tri-Directional Collaboration

The first theme spoke to the importance of joined-up working between professionals in the IOP and outpatient teams and parents to collaboratively support their young people to progress through treatment. Clinicians emphasised feelings of a shared responsibility and accountability that helped to activate the system around the child. This collaboration was an ever-changing process that demanded clear and consistent communication to prevent misalignment. The IOP served as a structure to strengthen and reinforce the therapeutic alliance between parents and clinicians, creating a tri-directional impact on trust, engagement, and outcome.

1a. All in it together

This sub-theme emphasised the feelings of shared responsibility that were experienced by the referring clinician in relation to their joint role with the IOP in catalysing change. Given both the complexity and physical risk associated with cases requiring IOP input, strong containment of the system around the child was required.

*‘And it helps with thinking together about what are the barriers to, I guess it can add into some of the formulation of the difficulties.’*

(Clinician 1)

Referring clinicians reported feeling supported by the IOP in managing such high levels of risk and described themselves as co-partners in a process, working alongside the IOP to facilitate progress.

*‘(The IOP) I think a big impact in a positive way because again, thinking about that kids journey—so they see (one clinician) immediately, see (a different clinician) on the ward and then their gone, and in a way the IOP team…. I think they were really helpful, actually in kind of holding everything together and then bringing me on board. There was this set up in a consistent way, with this family and in a sense…. I think they were quite instrumental.’*

(Clinician 2)

1b. Collaboration as a dynamic process

The collaboration between the IOP, referring clinician, and families was a dynamic process. The requirement for continuous negotiation and coordination between the three parties was emphasised by the referring clinicians. Flexibility, role clarity, and shared understanding were considered to foster alliance between all parties.

*‘There was a lot of joint work, and I just think it was fundamental for the outcomes of all these young people that we were able to work so closely together but also start to feel like they also can manage without my presence, and actually that’s still aligned with the goals that we had set.’*

(Clinician 9)

Clinicians reflected on the misalignment and communication breakdowns that resulted from reduced collaboration and the disruption to the therapeutic momentum that this could cause.

*‘They’d (the family) obviously formed relationships with the IOP team, which is great, and the ward staff. And then I was like this random person that would kind of pop up who, who didn’t, who because the IOP was understandably trying to keep me involved so that the family would have a relationship with me at the point that they were discharged, but then in the end they ended up staying there for so long. I was just a bit… They didn’t even know my name. They kept calling me X (wrong staff name). I think they were a bit confused about who I was.’*

(Clinician 6)

1c. Therapeutic alliance as a mechanism of change

There was reflection across referring clinicians of changes in the therapeutic alliance, a key mechanism of change, that occurred with IOP involvement. The IOP was generally considered to strengthen the alliance between parents and the referring clinician, which promoted a positive feedback loop whereby the alliance between the IOP and parents/referring clinician was further reinforced and vice versa.

*‘I don’t think what we were doing in outpatients stopped when the IOP took over and then it came back. So, it was more of a working together type of process. So, I think it was quite smooth, and I think that was the understanding of the family as well. It wasn’t that they were working with another team.’*

(Clinician 3)

Trust between all parties acted as the emotional glue holding the triad together with mutual reinforcement between the three points of this ‘triangle’ enhancing engagement and progress.

*‘I think that it helped improve engagement in that the family felt as though we were really responsive when they were stuck and were very flexible.’*

(Clinician 13)

#### 3.2.2. Creating Space for Change

The second theme described a core function of the IOP being in its ability to arrest rapid deterioration, creating both space and time for thoughtful planning about change. Clinicians considered this to be helpful—stabilisation created a base for containment and re-engagement with the therapeutic process, which may have been lost in the crisis. The bespoke nature of planning between referring clinicians, the IOP and families to individualise treatment was viewed as essential in planning effective treatment on a case-by-case basis, rather than as a one-size-fits-all approach.

2a. Pressing pause

Clinicians felt that the IOP team were integral in halting the speed of deterioration in order to prevent reactive decision-making.

*‘I think because they were much, much more aware of who she was and what her needs were, they provided what they needed to provide and when the young person was digging their heels in, they kind of gave her space. Just said, OK, well, we just keep reviewing, but still kind of kept to the plan that we’d agreed. So, they were much more focused and targeted and clear. We expect you to do this in these time frames. If not, then you know they would stop. We expect this, they would give the time and stop. And then at the end of the week, we had the evidence to look back on.’*

(Clinician 10)

This temporal slowing allowed for reflection and thoughtful planning. Metaphors of suspension and grounding dominated participants’ accounts.

*‘I think frequency was good because of the two face to face appointments that were happening during the week, I was joining them as well. So, there was a bit of a connect there. And we were able to see, you know, as a care coordinator… like you’re able to see what’s helping, what’s not helping and how you can support them.’*

(Clinician 7)

2b. A safety net

The IOP was described as a stabilising presence, especially in crises or uncertainty, both for referring clinicians and families. Their containment offered professionals and families a safety net to process complexity that was associated with young people referred to the IOP.

*‘Just that I think it is a good resource for us to have and it’s really valuable because we know what we can offer to families at time of, you know, crisis. This is first thing we do because we know hospitalisation doesn’t work and I mean it’s not always helpful … So I think the IOP is very, very important… to prevent things from deteriorating. And yeah, it’s good to just know that you have something to fall back on if you need that’*

(Clinician 4)

This “holding in suspension” created a platform for re-engagement with the therapeutic work.

*‘Yeah, I think it’s always helpful to have like an external team or more people involved in the case. Because you don’t always have time to get to know the young person and the family outside of the session. For example, if you are not there during mealtimes and there might be conversations that are helpful, that they (the IOP) can be there for and then they would pass on specific information for the formulation.’*

(Clinician 11)

2c. Bespoke treatment planning

Clinicians reflected on the importance of the IOP team adjusting their methods, pace, and focus according to the needs of each unique case. This adaptability was both logistical and relational, considered to be one way of meeting families where they were at this time of crisis.

*‘I think that can be in quite a flexible manner, which is what makes the IOP so useful. Often I think there’s a lot of meal support that is needed because young people are at risk of admission, so weight restoration and supporting with refeeding is often needed, but I think they can be really helpful in providing like support with distress tolerance, skills, emotion regulation, working with a young person’s motivation, doing some parent only work. I sort of felt like an extra set of hands was needed to intensify things because they were so stuck.’*

(Clinician 13)

The increased intensity of care mirrored the complexity of each case rather than rigidly adhering to protocol.

*‘We were able to make a good compromise about lunchtime and virtual support online, which felt like a good workaround. So, I felt like start took that into consideration about Mum’s kind of limited availability. Once we kind of got into that process, it was fine, I think and start we’re really accommodating about when and joining and stuff like that.’*

(Clinician 8)

#### 3.2.3. Transitions as Turning Points

Theme three was centred around endings. Referring clinicians were reflective about the nature of IOP ending, whereby the road ahead remained unclear despite feeling more settled. Views from referring clinicians were split, with some viewing the end of the IOP as a valuable time for treatment review, grasping this as an opportunity to navigate a new way forward with their families. Others felt uncertain and uncontained by the ending of the IOP, desiring more time to continue the collaborative working and develop a road map for ongoing treatment together.

3a. Endings as opportunities for reflection and review

The conclusion of the IOPs involvement invited reflection on what had changed and what remained needed by all referring clinicians that were interviewed as part of this study. The review processes helped to solidify learning, strengthen relationships, and for some, identify sustainable next steps.

*‘It is a good opportunity when the IOP completes their input and they stop being involved. It’s a good opportunity for a review of the needs overall, review of what the family has learnt—what they need to keep on doing in order to keep the momentum going and a review of the goals for later states of the treatment in outpatient.’*

(Clinician 5)

The end of IOP was considered by some clinicians as a reflective pause and a chance to recalibrate before moving forward.

*‘The IOP was going to work with them for a short time and then we might be thinking about (the day program), … because we have like a transition to (the day program), it kind of works because we are doing the intensification from the IOP to (the day program) so that feels like the IOP is like a good transition in some way’*

(Clinician 11)

3b. Living with uncertainty: ‘What happens next?’

Some referring clinicians experienced overwhelming uncertainty around the next stage of care. Referring clinicians’ ambiguity about their next steps after the IOP was navigated differently across those interviewed. A trend was observed between clinician level of confidence and the tolerance of uncertainty regarding ongoing treatment. Clinicians who reported feeling more confident about steering the treatment direction post-IOP were similarly keen to embrace the uncertainty as an opportunity for change.

*‘I can’t think of anything that was unhelpful because I think everybody realized that actually for this family, too many cooks are going to spoil the broth. They needed a consistent message, they needed to build a therapeutic relationship with someone they felt contained with and I think that they felt they, now they’re building a relationship with this person, and all the decisions are going to be made with that person and not the IOP.’*

(Clinician 2)

Those who felt less hopeful about the transition away from the IOP felt less confident in navigating the uncertainty of the next steps.

*‘So yes, sometimes I feel that there’s a lot of thinking around the case, but not so many very clear 1-2-3 steps. Thinking about what needs to be done while at the same time it’s known that we only have four weeks, we need to come up with something.’*

(Clinician 15)

3c. Clinician confidence as a source of containment

The clinician’s ability to project confidence, stability, and clarity helped to contain the system during transitions. Referring clinician confidence acted as a psychological anchor, maintaining trust and belief in ongoing progress post-IOP.

*‘I know that the IOP can provide a limited time of support and then you’re like we have this deadline to come up with a very, very clear plan and sometimes outpatient don’t give this clarity on time … we need to be very clear because I want to allow the families a good amount of time to prepare themselves for the next step… Especially because mainly all of my patients are also autistic… and I feel safer if I have very, very clear instructions.’*

(Clinician 15)

When this containment was present, those interviewed in this study reflected on their families being better able to internalise a sense of safety and continuity.

*‘One of the goals that we didn’t manage to achieve with that process is that it would be a sustained change that meant we could continue just with outpatients, which was not the case because the need was greater than that. But I think at the same time, it was a helpful stepping stone to getting people more ready, more able and willing to be able to engage in a more long-term intensive treatment.’*

(Clinician 2)

## 4. Discussion

This study explored the experiences of referring clinicians working alongside an IOP for children and adolescents with EDs and generated three key themes: (1) Tri-directional Collaboration, (2) Creating Space for Change, and (3) Transitions as Turning Points. The findings highlight the value of the IOP as a complementary component within the current care pathway, while also identifying important challenges and opportunities for development.

Clinicians consistently described the IOP as enhancing collaboration between outpatient teams, families, and the young person. Interestingly, although participating clinicians spanned a diverse range of professional backgrounds—they shared similar experiences from working with the IOP. All clinicians were both the lead therapist and care coordinator in the case of each patient they referred to the IOP (i.e., all were providing the course of therapy rather than being involved solely to fulfil the duties of their specific profession). The sense of togetherness signified a shared responsibility for risk management, treatment direction, and progress. Collaboration was understood as dynamic, requiring clear communication, negotiated roles, and adaptive coordination. When effective, this process strengthened the therapeutic alliance across all relational points, between families and outpatient clinicians, between families and the IOP, and between clinicians themselves. This tri-directional alliance functioned as a mechanism of change, supporting trust, engagement, and coherence across the system.

These findings align with broader literature demonstrating that interprofessional coordination and relational consistency are essential in health care (Bray et al., 2025; Reeves et al., 2017). Studies of other intensive community programmes, such as day programmes, similarly identify joint working, shared formulations, and relational containment as central to positive outcomes (Baudinet & Simic, 2021; Krishnamoorthy et al., 2023; Webb et al., 2022; White et al., 2025). This triangulation suggests that the collaborative ethos of the IOP may be one of its most critical mechanisms of action. Similarly, consistency, containment, and collaboration have been highlighted as core components by parents and young people who experienced working with an IOP during their ED treatment (Brennan et al., 2026).

Clinicians experienced the IOP as providing essential containment during periods of rapid deterioration. The programme’s intensity and frequency created space for a pause; slowing the pace of crisis, allowing for more reflective decision-making, and providing a safety net that helped families and clinicians manage complexity. This stabilising function aligns with evidence from adolescent day programme models, where intensive support has been shown to improve weight, reduce eating disorder symptoms, and prevent or shorten inpatient admissions (Ali et al., 2021; Baudinet et al., 2020; Simic et al., 2018).

Another core strength identified was the IOP capacity for bespoke, case-by-case adaptation. Clinicians valued the programme’s ability to adjust the pace and focus of intervention, whether through meal support, parent-only sessions, or targeted motivational approaches. This flexibility was perceived as essential for meeting families where they were, particularly when working with comorbidities, neurodiversity, entrenched patterns, or heightened distress. Patient-centred and individualised care resonates with recommendations advocating for personalised, needs-led models of intensive community care (Baudinet et al., 2021a; Bryant et al., 2025). It also fits with qualitative data from first-line outpatient treatments, where engaging the young person and parents is essential for enabling change (Baudinet et al., 2024; James et al., 2025).

Endings emerged as pivotal moments that shaped clinicians’ overall experience of the IOP. The conclusion of the IOP served as an opportunity for reflection and consolidation. The time-limited nature of the programme allowed clinicians and families to review progress and establish a renewed direction for ongoing treatment. However, IOP endings also evoked uncertainty, particularly when post-IOP plans were unclear or when the family had become reliant on the structure and intensity the programme provided.

### Limitations

Limitations of our findings include the single-site design which may limit the transferability of findings to other services and systems. This limitation was partially mitigated by the inclusion of a diverse range of clinicians. Future research should explore the experiences of clinicians from multiple sites. The patient/carer perspectives are not contained within this article—although explored in another parallel publication on the same IOP. The lack of longitudinal clinician follow-up limits the ability to relate our findings to longer term treatment outcomes for IOP interventions, ongoing data collection will support future longitudinal investigation. Further research should examine the mechanisms by which IOPs exert their effects, including the relative contributions of intensity, relational work, parental engagement, and systemic coordination. The role of IOPs for young people transitioning to adult ED services was not investigated in this study and should be explored in further research to understand the potential contribution to care.

## 5. Conclusions

Embedding IOPs within stepped-care frameworks may represent an effective and scalable means of expanding system capacity while providing enhanced, flexible support during periods of heightened risk (Knight et al., 2025). However, longitudinal, mixed-methods evaluations are needed to clarify the sustainability of progress post-IOP and identify predictors of positive transitions.

## Figures and Tables

**Table 1:** Comparison of IOP, day programme/partial hospitalisation programme, residential, and inpatient settings for ED—similarities and differences.

	IOP	Day Programme/Partial Hospitalisation Programme	Residential	Inpatient
Professionals	Multi-disciplinary	Multi-disciplinary	Multi-disciplinary	Multi-disciplinary
Interventions	Psychosocial, nutritional, medical	Psychosocial, nutritional, medical	Psychosocial, nutritional, medical	Psychosocial, nutritional, medical
Modality	Individual care	Individual and group-based care	Individual and group-based care	Individual and group-based care
Setting	Community and paediatric wards	Community	Residential unit	Inpatient ward
Duration	~2–6 weeks	~3–18 weeks	~11–12 weeks	>12 weeks
Frequency	4 to 7 days per week	4 to 7 days per week	24 h/day, 7 days per week	24 h/day, 7 days per week

**Table 2:** Themes and sub-themes generated.

Themes	Subthemes
1. Tri-directional Collaboration	1a. All in it together
	1b. Collaboration as a dynamic process
	1c. Therapeutic alliance as a mechanism of change
2. Creating Space for Change	2a. Pressing pause 2b. A safety net 2c. Bespoke treatment planning
3. Transitions as Turning Points	3a. Endings as opportunities for reflection and review 3b. Living with uncertainty: ‘What happens next?’ 3c. Clinician confidence as a source of containment

## Data Availability

The datasets presented in this article are not readily available because the data are part of an ongoing study. Requests to access the datasets should be directed to the corresponding author.
